# Generative artificial intelligence: synthetic datasets in dentistry

**DOI:** 10.1038/s41405-024-00198-4

**Published:** 2024-03-01

**Authors:** Fahad Umer, Niha Adnan

**Affiliations:** Operative Dentistry and Endodontics, Department of Surgery, Aga Khan University Hospital, Karachi, Pakistan

**Keywords:** Digital radiography in dentistry, Health care

## Abstract

**Introduction:**

Artificial Intelligence (AI) algorithms, particularly Deep Learning (DL) models are known to be data intensive. This has increased the demand for digital data in all domains of healthcare, including dentistry. The main hindrance in the progress of AI is access to diverse datasets which train DL models ensuring optimal performance, comparable to subject experts. However, administration of these traditionally acquired datasets is challenging due to privacy regulations and the extensive manual annotation required by subject experts. Biases such as ethical, socioeconomic and class imbalances are also incorporated during the curation of these datasets, limiting their overall generalizability. These challenges prevent their accrual at a larger scale for training DL models.

**Methods:**

Generative AI techniques can be useful in the production of Synthetic Datasets (SDs) that can overcome issues affecting traditionally acquired datasets. Variational autoencoders, generative adversarial networks and diffusion models have been used to generate SDs. The following text is a review of these generative AI techniques and their operations. It discusses the chances of SDs and challenges with potential solutions which will improve the understanding of healthcare professionals working in AI research.

**Conclusion:**

Synthetic data customized to the need of researchers can be produced to train robust AI models. These models, having been trained on such a diverse dataset will be applicable for dissemination across countries. However, there is a need for the limitations associated with SDs to be better understood, and attempts made to overcome those concerns prior to their widespread use.

## Introduction

Digitization of healthcare has enabled Artificial Intelligence (AI) methods to integrate big data for effective patient management [1]. AI models particularly deep learning algorithms such as Convolutional Neural Networks (CNNs), vision transformers, and Large Language Models (LLMs) require large amounts of training data to achieve acceptable performances [2–5]. This has increased the demand for digital data in all domains of healthcare, including dentistry [6]. Healthcare data available for training AI models includes Electronic Health Records (EHRs), radiographs, clinical photographs, etc [1]. This extensive patient data can be curated to form large-scale datasets for training AI on healthcare-related problems.

However, some pertinent concerns prevail with AI models trained on these “traditionally acquired” datasets. An obvious hurdle is that of patient privacy that makes dataset acquisition difficult for researchers [7]. Other problems are those of representation of populations; due to unavailability of widespread data, some communities may remain under-represented. AI models trained on such datasets are not generalizable to the global population. For example, a widely used AI algorithm in healthcare underestimated the treatment needs of African Americans and failed to triage them for necessary care due to a lack of sufficient data instances [8]. Another facial recognition software was biased towards Caucasians and failed to identify dark-skinned individuals [9]. Moreover, healthcare data is in a constant state of “shift” due to changes in medical practices as well as patient behavior requiring constant upgrade of data to ensure the real-time applicability of an AI model trained on this information [10]. Therefore, there is a need for dynamic and representative datasets to ensure the universal applicability of AI models [11].

One possible solution is the production of “synthetic” heterogenous datasets by using generative AI [12]. Recently, interest in generative AI has amplified since the advent of LLMs such as ChatGPT (OpenAI) and diffusion models such as DALL-E (OpenAI) and Midjourney (Midjourney Inc.) [5, 13, 14]. If translated into healthcare, this technique holds great promise for overcoming challenges associated with the need for diverse and inclusive datasets without privacy concerns [15]. Generative AI techniques can be streamlined into research to develop broad-ranging Synthetic Datasets (SDs) that simulate real-life healthcare data. These generative models learn patterns from the input (training data) and produce synthetic data as outputs [15].

The following narrative describes challenges faced by researchers in the curation of traditional datasets. It further explains the mechanism of some generative AI techniques that can be used to generate SDs.

## Challenges of “traditionally acquired” datasets

### Data privacy and ethical concerns

On an organizational level, healthcare data is costly and is associated with a deep-seated resistance to data sharing between institutions since it is considered hospital property [2]. Data transfer agreements, including the Portability and Accountability Act of 1996 in the United States, have set strict regulations to ensure patient confidentiality [15]. These regulations make it exceedingly difficult for researchers to access medical data, leading to a lack of diversity in the currently available datasets. Additionally, erasing the metadata associated with medical images does not ensure patient privacy, which can still be compromised, making it challenging to share data across institutions [15].

### Data annotation

The curation and annotation of large-scale datasets is a time-consuming and resource-intensive task [3]. It requires subject experts to manually label all features in an image, spending valuable time developing the dataset. This cumbersome task prevents researchers from investing time in performing experiments [3, 11]. Moreover, errors can be incorporated into these labels, decreasing the accuracy of the AI model trained on this data. The generation of synthetic and fully annotated data can potentially eliminate laborious annotations, accelerating AI research.

### Biases

Bias may be quantified as the differential impact of a healthcare process on a particular subgroup [16]. AI may be considered free of human bias due to its self-learning nature, however, biases in AI development can be introduced at any stage of the algorithm development process [16]. This is due to the black-box nature of contemporary AI systems [17].

Due to the upsurge of AI in developed countries, the datasets produced are not representative of the global population, which has led to data-driven biases such as those of socioeconomic standing and ethnicity [18]. Another type of bias that may be incorporated into traditional datasets is due to the acquisition of images from a specific machine. All images in this dataset have distinct dimensions limiting variations in data required to develop a generalizable AI model. Moreover, the presence of “class imbalances” in datasets, such as the lack of sufficient examples of a rare disease in dataset gathered from a population, inculcates sample selection bias that also hinders the real-life applicability of AI models trained on these datasets [18]. It is therefore imperative that data from as many countries as possible be included.

## Generative AI techniques

There are various AI techniques for generating synthetic datasets. The mechanisms of more common ones have been described in the following text.

### Variational Autoencoders (VAEs)

**Fig. 1:**
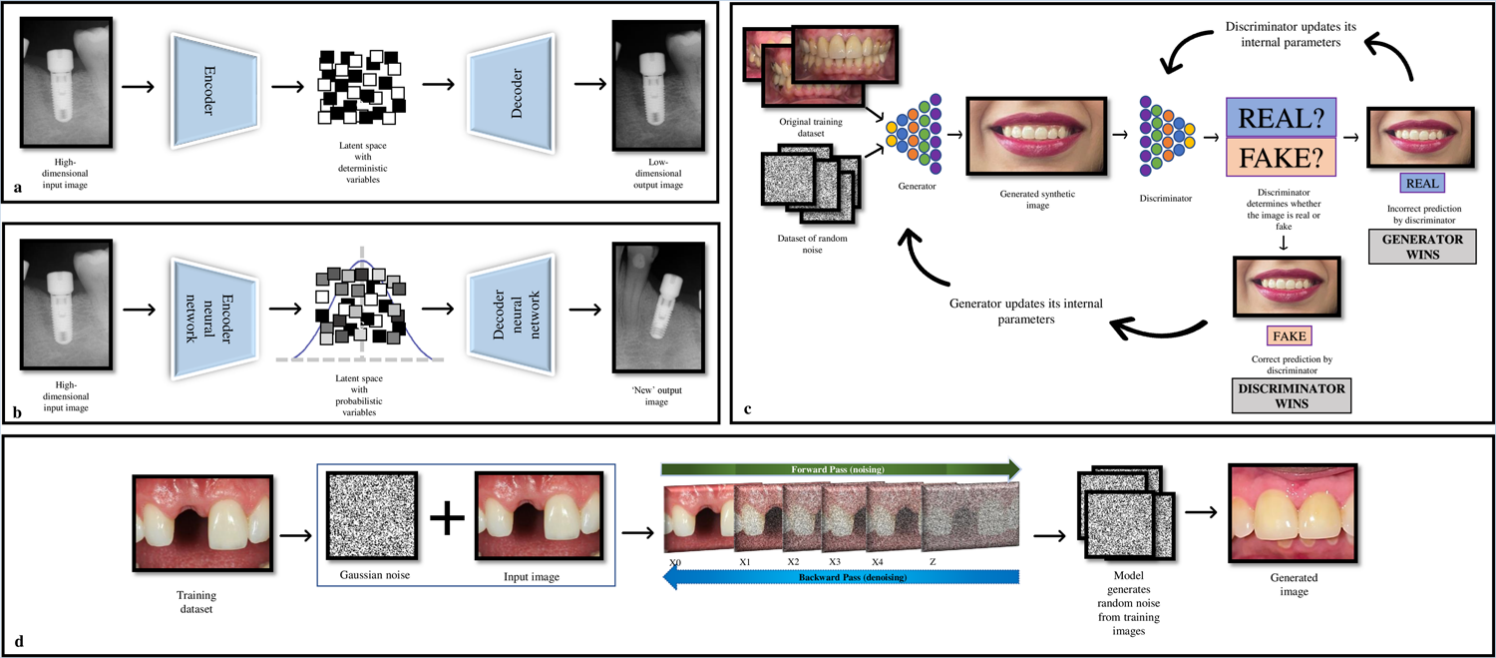
Generative AI techniques. **a** Auto Encoder—A high-dimensional input image processed through the latent space with deterministic variables to produce a low-dimensional output image. **b** Variational Autoencoder—A high-dimensional input image processed through the latent space with probabilistic variables to produce a “new” high-dimensional output image. **c** The workflow of a generative adversarial network, showing the working of generator and discriminator models. Incorrect predictions lead to the generator and discriminator model adjusting their internal parameters to improve their performance after each iteration. **d** The forward and backward passes of a diffusion model which uses Gaussian noise to generate a “new” image.

VAEs are an advanced version of a deep generative model known as Auto Encoder (AE), that can learn complicated patterns in training data. AE consists of two parts: an “encoder” and a “decoder”; the encoder takes high-dimensional input data, such as an image or a text sequence, and maps it to a lower-dimensional representation known as “latent space”. This space is a compressed version of original input data, capturing the most relevant features in a simplified form for easier processing [19, 20]. The decoder then employs this compressed latent space data and reconstructs it back to its original form. However, AEs are deterministic, meaning that no “new’ data is generated, and these can only reconstruct the original data [19, 20] (Fig. 1a, b).

VAEs extend the concept of AEs by introducing statistical techniques, such as probability distributions (a mean value and standard deviation), into the latent space. This probabilistic approach allows VAEs to learn a range of possible values for each encoded input, meaning that VAEs can reconstruct original data by creating new and similar data points of the original data, leading to the production of synthetic data [21, 22].

VAEs have been used in medicine and dentistry, enabling the generation of realistic medical images and health records, improving lung sound classification, and overcoming data limitations in medical imaging analysis. Their effectiveness in generating clinical sounds, images, and realistic EHRs shows promise in advancing data-driven medical care delivery [23].

### Generative Adversarial Networks (GANs)

GANs comprise two neural networks: a “generator” and a “discriminator” [20, 24]. The generator uses random noise (gaussian) and produces an image which closely resembles the real images. The discriminator receives the images with the task of distinguishing between “real” and “fake” ones [20]. As the name suggests, these models are trained in an adversarial way; the generator is trained to generate images closely mimicking real images to mislead the discriminator. On the other hand, the discriminator is educated to progressively improve at distinguishing real images from the false ones. The generator improves at generating realistic images as the training goes on, while the discriminator improves at detecting fraudulent ones. In this concept there is always a winner and loser in which the loser will update its internal parameters for the next iteration, thus ensuring constant improvement (Fig. 1c).

Researchers have used GANs to generate two-dimensional cephalograms from Cone Beam Computed Tomography and improve landmark detection [25]. GANs have been utilized to generate highly realistic intraoral images which experienced pediatric dentists were unable to distinguish as either fake or real [26].

### Diffusion models

Recently, diffusion models have gained popularity as generative AI that can yield superior results compared to GANs and VAEs [27] (Fig. 1d). These models create new data using a two-step process: the “forward pass” (encoder) and the “backward pass” (decoder). During the forward pass, noise (gaussian) is progressively added to the data (pictures, radiographs, etc) in an iterative manner. This means that at each step of the process, noise is introduced into the image. In the backward pass, the model reverses this process and attempts to remove noise from the image. This allows the model to learn the relationship between different pixels in an image. Thus, the models “corrupt” training image by adding noise and then learn to recover the data during the denoising process. The model can therefore be trained to generate new images using prompts [27].

In healthcare research, diffusion models have been utilized to generate realistic chest X-ray, Magnetic Resonance Imaging and histology images [28].

## Chances of SDs

SDs can add further value to the following healthcare domains.

### Research

Since the performance of an AI model is dependent on the amount and variation of data used for training, SDs can lead to the development of robust models [3]. Traditionally, data augmentation is carried out by simple modifications to the training dataset such as flipping, rotation, translation, etc but these alterations add limited new features to the dataset [29]. The generation of synthetic images allows for augmentation at a wider scale by adding images of greater variance to the dataset.

The potential of generative AI is being considered for producing new types of data for researchers to conduct experiments. For example, in drug discovery, where AI can be utilized to generate candidate molecules for pharmaceuticals to create new compounds for lab testing [30]. Synthetic Minority Oversampling Technique (SMOTE) can be used where specific rare examples of disease can be generated and purposefully combined into SDs to include all possible features of data [31]. SMOTE can also potentially mitigate “shifts” in data, by adding synthetic examples of new emerging data features within populations. This will ensure that the SDs remain applicable and generalizable with the constantly changing medical practices [32].

### Education

SDs can also be developed for creating simulations for training dental and medical students [1]. These simulations can be customized to the individual needs of every student, helping train them in their areas of deficiency [33]. With the current pace of advancements in generative AI, a virtual instructor with generated characters seems likely in the near future. Educational chatbots specific to different fields of study are already emerging, with sure improvements in the future.

## Challenges of SDs

The extensive use of SDs is not without its shortcomings which may have a negative impact on patient care.

### Confidentiality

Due to its ability to learn the original distribution of data, generative AI may still lead to patient re-identification by associating the image with information such as patient visit date and time of exposure [23]. Therefore to mitigate this issue, any patient identifiers need to be manually removed from the training images to prevent “privacy leakage” [23].

### Metrics for “syntheticism”

There is a lack of research relating to the evaluation of these generative AI models. Standardized evaluation metrics are needed to determine the level of syntheticism of SDs [34]. The images should be “synthetic” enough to avoid any resemblance to real images, and “realistic” enough to be applicable to real-world scenarios. Before SDs can be employed for training AI, its realism needs to be determined by a subject expert [1, 35]. Moreover, it has been shown that synthetic images of lower resolution are difficult to distinguish and exhibit more “realism” than those with higher resolutions [26]. One such metric for evaluating the realism of SDs include “fidelity” that measures the structural similarity between original and synthetic images [36].

### Biases

**Fig. 2:**
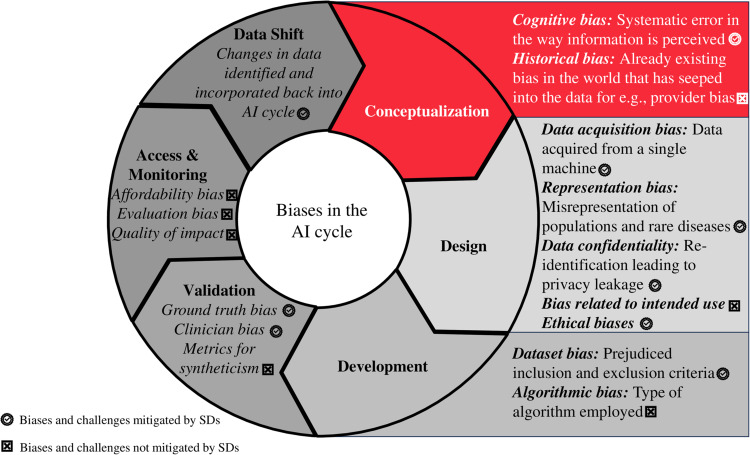
Challenges and biases in the entire AI cycle. Denoted are the aspects that can be managed with the use of SDs.

Despite the advantage of reduced biases associated with SDs, generative models can still retain some of the biases [36]. Since humans are involved in the production of synthetic data, the inherent risk of incorporating human bias remains an area of concern. Similar to “provider bias” where a physician may be prejudiced against a certain racial group for example, these biases invariably translate into AI models [36]. If these biases are not addressed at an early stage of AI development, it can lead to inaccurate results that are not representative of the population [37]. For the elimination of biases, it is crucial to identify all possible biases as well as the cause of their incorporation during the “AI cycle” (Fig. 2). One method includes the bioethical analysis of AI cycles from the initial developmental stages through deployment [36]. The identification and mitigation of biases improve “AI fairness”; this implies the equal performance of the AI model across all subgroups in a population [38]. Some measures to ensure clinical AI fairness include panels of AI researchers, clinicians, and ethicists proactively overseeing the process of dataset curation and model training to ensure a clinically fair AI model [32]. Detailed discussions on mitigation of biases in AI is beyond the scope of this paper and can be found elsewhere [36].

### False synthetic data

Generative AI can purposefully be used for the production of false data also known as “deepfakes” [39]. These synthetically altered images are a threatening advancement in AI that can potentially cause harm to individuals as well as industries [39].

Hallucination is a pertinent issue that has surfaced with the widespread use of generative AI. This refers to the phenomenon of production of synthetic images with factual errors, much like the incorrect information that has been produced by ChatGPT [39]. Since synthetic healthcare data is representative of authentic data that is used to treat humans, the incorporation of data with errors such as incorrect anatomy or false illustration of disease can be detrimental. Further research is required to better understand the implication of hallucinations and deepfakes in synthetic medical and dental data. The reliability of SDs is difficult to determine as there are no evaluation metrics so far since this aspect of AI is still under development. One possible way to determine the robustness of SDs is to involve subject-level experts such as doctors for medical images, to validate the accuracy of the images before utilization and is an area of future research.

## Conclusion

VAEs, GANs and diffusion models have been utilized in healthcare research to generate SDs. Synthetic data customized to the need of researchers can be produced to train robust AI models. These models, having been trained on such a diverse dataset will be applicable for dissemination across countries. However, there is a need for the limitations associated with SDs to be better understood, and attempts made to overcome those concerns prior to their widespread use. Of greater importance is the need for AI governance to ensure that generative AI is being implemented in a way that is beneficial to society [40]. Transparency in AI development and adherence to strict standards is a prerequisite for this technology to make a noticeable impact in healthcare settings.

## Data Availability

Data availability statement is not applicable to this study since no research data was used.
